# Supplementary material for: An ecological study to evaluate the association of Bacillus Calmette-Guerin (BCG) vaccination on cases of SARS-CoV2 infection and mortality from COVID-19
Source: PLoS One. 2020 Dec 17;15(12):e0243707. doi: 10.1371/journal.pone.0243707 (PMC7746266; doi:10.1371/journal.pone.0243707)
Supplement: S1 Table — (DOCX) [file pone.0243707.s001.docx]

**S1 Table: Morbidity and mortality from SARS-CoV2 pandemic and the population, economic, and health characteristics of selected countries with a current BCG policy**

| Country | **POPULATION INDICATORS** | | **COVID-19 INDICATORS** | | | **ECONOMIC INDICATORS** | | | **HEALTH INDICATORS** | | |
| --- | --- | --- | --- | --- | --- | --- | --- | --- | --- | --- | --- |
|  | Population size^1^ | Population >65 years^2^ (%) | Tests per capita^3^ (n) | Confirmed cases^4^ (n) | Fatal cases^5^ (n) | Gross domestic product ($)^6^ | Income level^7^ | Net Immigration^8^ | Smoking prevalence (%) | Stringency levels at 100 cases | Stringency levels 28 days after 10^th^ fatal case |
| Republic of Korea (South) | 51 258 252 | 14 | 20 810 | 11 468 | 270 | 1 619 | High | 58 657 | 25.35 | 31.35 | 75.93 |
| Japan | 126 547 906 | 28 | 2 567 | 16 851 | 891 | 4 971 | High | 357 800 | 19.20 | 21.03 | 45.37 |
| Singapore | 5 841 107 | 11 | 83 569 | 34 366 | 23 | 364 | High | 135 142 | 17.07 | 23.41 | 81.48 |
| Portugal | 10 202 661 | 22 | 93 553 | 32 203 | 1 396 | 241 | High | - 30 001 | 21.24 | 46.82 | 82.41 |
| Ireland | 4 928 757 | 14 | 74 531 | 24 929 | 1 651 | 383 | High | 118 020 | 19.90 | 50.26 | 90.74 |
| Poland | 37 855 780 | 18 | 29 830 | 23 571 | 1 061 | 586 | High | -146 976 | 24.58 | 60.05 | 83.33 |
| Greece | 10 432 358 | 22 | 23 109 | 2 915 | 175 | 218 | High | - 80 000 | 39.76 | 33.33 | 84.26 |
| Croatia | 4 109 782 | 20 | 16 950 | 2 246 | 103 | 61 | High | - 40 004 | 37.64 | 74.61 | 89.81 |
| Hungary | 9 665 208 | 19 | 23 003 | 3 867 | 524 | 158 | High | 29 999 | 25.23 | 69.18 | 74.07 |
| Estonia | 1 326 603 | 20 | 70 741 | 1 865 | 67 | 31 | High | 19 555 | 30.50 | 56.35 | 67.13 |
| Lithuania | 2 728 924 | 20 | 128 777 | 1 670 | 70 | 53 | High | -163 902 | 28.10 | 81.48 | 77.78 |
| Latvia | 1 890 028 | 20 | 65 370 | 1 065 | 24 | 34 | High | -74 186 | 34.46 |  |  |
| Saudi Arabia | 34 707 744 | 3 | 29 321 | 83 384 | 480 | 787 | High | 674 895 | 19.35 | 52.38 | 94.44 |
| United Arab Emirates | 9 869 809 | 1 | 261 244 | 33 896 | 262 | 414 | High | 200 000 | 0.00 | 41.14 | 81.48 |
| Qatar | 2 870 383 | 1 | 97 868 | 55 262 | 36 | 191 | High | 200 000 | 0.00 | 45.64 | 87.04 |
| Kuwait | 4 257 166 | 3 | 76 672 | 26 192 | 205 | 141 | High | 197 600 | 0.00 | 71.83 | 100 |
| Oman | 5 080 768 | 2 | 25 407 | 10 423 | 42 | 79 | High | 437 000 | 17.14 | 83.6 | 84.26 |
| Chile | 19 088 491 | 12 | 39 833 | 94 858 | 997 | 298 | High | 558 539 | 35.82 | 44.31 | 75.93 |
| Uruguay | 3 471 541 | 15 | 14 533 | 816 | 22 | 60 | High | -15 000 | 18.72 | 60.44 |  |
| Afghanistan | 38 757 826 | 3 | 1 352 | 15 094 | 250 | 19 | Low | -314 602 | 0.00 | 61.25 | 78.7 |
| Burkina Faso | 20 794 141 | 2 |  | 853 | 53 | 14 | Low | -125 000 | 22.70 | 88.23 | 84.26 |
| Senegal | 16 660 525 | 3 | 3 328 | 3 535 | 42 | 24 | Low | -100 001 | 12.95 | 77.78 | 72.22 |
| Democratic Republic of Congo | 89 045 192 | 3 |  | 2 965 | 68 | - | Low | 119 303 | 35.85 | 97.22 | 80.56 |
| Philippines | 109 304 469 | 5 | 4 229 | 17 224 | 950 | 331 | Lower middle | -335 758 | 23.20 | 83.33 | 97.22 |
| Vietnam | 97 177 281 | 7 | 2 827 | 328 | - | 245 | Lower middle | -399 999 | 23.65 | 48.41 |  |
| Cambodia | 16 675 724 | 5 | 1 492 | 125 | - | 25 | Lower middle | -149 999 | 21.84 |  |  |
| Ukraine | 43 781 361 | 16 | 10 435 | 23 672 | 708 | 131 | Lower middle | 50 001 | 28.59 | 92.06 | 88.89 |
| Uzbekistan | 33 379 954 | 4 | 22 385 | 3 554 | 14 | 51 | Lower middle | -44 314 | 12.50 | 92.2 | 86.11 |
| Armenia | 2 962 149 | 11 | 25 444 | 9 282 | 131 | 12 | Lower middle | -24 989 | 23.44 |  |  |
| Kyrgyzstan | 6 504 219 | 4 | 21 420 | 1 748 | 16 | 8 | Lower middle | -20 000 | 26.33 | 87.31 | 79.17 |
| India | 1 377 458 927 | 6 | 3 780 | 182 143 | 5 164 | 2 719 | Lower middle | - 2 663 434 | 9.52 | 28.97 | 96.3 |
| Indonesia | 272 979 746 | 6 | 1 696 | 25 773 | 1 573 | 1 042 | Lower middle | - 494 777 | 42.85 | 45.36 | 66.2 |
| Sri Lanka | 21 396 349 | 10 | 3 818 | 1 620 | 10 | 89 | Lower middle | - 489 932 | 13.59 | 71.83 |  |
| Pakistan | 220 089 716 | 4 | 3 539 | 69 496 | 1 483 | 315 | Lower middle | - 1 166 895 | 23.42 | 52.78 | 89.81 |
| Egypt | 101 971 164 | 5 | 1 321 | 23 449 | 913 | 251 | Lower middle | - 190 164 | 28.81 | 7.94 | 84.26 |
| Morocco | 36 828 518 | 7 | 9 878 | 7 780 | 204 | 118 | Lower middle | - 257 096 | 26.15 | 81.09 | 90.74 |
| Tunisia | 11 796 383 | 8 | 4 759 | 1 076 | 48 | 40 | Lower middle | - 20 000 | 0.00 | 75 | 87.96 |
| Côte d’Ivoire | 26 256 464 | 3 | 1 271 | 2 799 | 33 | 43 | Lower middle | -40 000 | 0.00 | 76.73 | 37.04 |
| Ghana | 30 950 462 | 3 | 7 740 | 7 768 | 35 | 66 | Lower middle | - 50 000 | 7.80 | 44.44 | 62.04 |
| Nigeria | 205 169 512 | 3 | 403 | 9 855 | 273 | 397 | Lower middle | - 300 000 | 10.74 | 75.55 | 80.56 |
| Bangladesh | 164 383 248 | 5 | 2 779 | 44 608 | 610 | 0 | Lower middle | -1 847 503 | 17.64 | 87.04 | 89.81 |
| Cameroon | 26 420 650 | 3 |  | 5 659 | 185 | 39 | Lower middle | - 24 000 | 33.40 | 53.96 | 63.89 |
| China | 1 438 314 867 | 11 |  | 84 570 | 4 645 | 13 608 | Upper middle | -1 741 996 | 23.99 | 0 | 75.46 |
| Malaysia | 32 289 159 | 7 | 19 120 | 7 762 | 115 | 359 | Upper middle | 249 999 | 21.04 | - | 73.15 |
| Turkey | 84 172 983 | 8 | 29 087 | 163 103 | 4 515 | 771 | Upper middle | 1 419 610 | 22.41 | 53.58 | 78.7 |
| Russian Federation | 145 925 086 | 15 | 95 080 | 405 843 | 4 693 | 1 658 | Upper middle | 912 279 | 38.76 | 44.31 | 85.19 |
| Romania | 19 260 123 | 18 | 27 625 | 19 133 | 1 253 | 240 | Upper middle | - 369 997 | 27.08 | 58.46 | 87.04 |
| Belarus | 9 450 007 | 15 | 71 810 | 41 658 | 229 | 60 | Upper middle | 43 648 | 24.79 | 8.33 | 13.89 |
| Moldova | 4 035 673 | 4 | 15 697 | 8 098 | 294 | 5 | Upper middle | 56 851 | 25.75 | 71.16 | 80.56 |
| Kazakhstan | 18 734 492 | 7 | 55 964 | 10 858 | 38 | 179 | Upper middle | - 90 000 | 23.87 | 70.28 | 89.35 |
| Azerbaijan | 10 122 210 | 6 | 35 132 | 5 246 | 61 | 47 | Upper middle | 6 002 | 21.85 | 60.72 | 85.19 |
| Bosnia and Herzegovina | 3 285 197 | 16 | 22 664 | 2 493 | 152 | 20 | Upper middle | - 107 926 | 36.34 | 94.05 | 90.74 |
| Albania | 2 878 618 | 14 | 6 017 | 1 136 | 33 | 15 | Upper middle | - 69 998 | 28.48 | 84.39 | 87.96 |
| Georgia | 3 990 877 | 15 | 17 688 | 783 | 12 | 18 | Upper middle | - 50 000 | 29.74 | 100 | 69.44 |
| Bulgaria | 6 958 023 | 21 | 13 785 | 2 513 | 140 | 65 | Upper middle | - 24 001 | 31.51 | 58.6 | 73.15 |
| Thailand | 69 767 610 | 12 | 6 708 | 3 081 | 57 | 505 | Upper middle | 97 222 | 20.52 | 27.25 | 81.02 |
| Iran | 83 797 576 | 6 | 13 978 | 148 950 | 7 734 | 454 | Upper middle | -274 998 | 10.09 | 20.9 | 51.85 |
| Iraq | 40 043 486 | 3 | 8 229 | 6 179 | 195 | 224 | Upper middle | 39 171 | 0.00 | 77.12 | 96.3 |
| Jordan | 10 180 078 | 4 | 23 578 | 734 | 9 | 42 | Upper middle | 51 099 | 46.93 | 94.58 |  |
| Brazil | 212 280 721 | 9 | 6 422 | 465 166 | 27 878 | 1 869 | Upper middle | 106 000 | 13.23 | 45.78 | 74.54 |
| Peru | 32 892 482 | 8 | 38 117 | 148 285 | 4 230 | 222 | Upper middle | 495 345 | 2.50 | 96.03 | 94.44 |
| Ecuador | 17 594 476 | 7 | 7 271 | 38 571 | 3 334 | 108 | Upper middle | 182 000 | 7.55 | 45.37 | 93.52 |
| Mexico | 128 678 447 | 7 | 2 866 | 84 627 | 9 415 | 1 221 | Upper middle | - 300 000 | 11.18 | 8.33 | 82.41 |
| Dominican Republic | 10 827 727 |  | 9 227 | 16 908 | 498 | 86 | Upper middle | -150 000 | 13.15 | 25 | 85.19 |
| Colombia | 50 790 443 | 8 | 8 750 | 26 688 | 853 | 331 | Upper middle | 1 023 981 | 9.51 | 48.28 | 90.74 |
| Argentina | 45 118 176 | 11 | 4 616 | 14 702 | 510 | 520 | Upper middle | 24 000 | 20.39 | 88.89 | 100 |
| Cuba | 11 327 642 | 15 | 11 031 | 2 025 | 83 | 100 | Upper middle | - 72 000 | 33.90 | 49.61 | 85.19 |
| Costa Rica | 5 085 430 | 10 | 5 965 | 1 022 | 10 | 60 | Upper middle | 21 000 | 11.85 | 74.61 | - |
| Venezuela | 28 427 319 | 7 | 37 040 | 1 370 | 14 | 482 | Upper middle | - 3 266 243 | 0.00 | 84.26 | 82.41 |
| South Africa | 59 168 133 | 5 | 16 846 | 30 967 | 643 | 368 | Upper middle | 727 026 | 17.55 | 57.93 | 84.26 |
| Mauritius | 1 271 426 | 11 | 108 354 | 335 | 10 | 14 | Upper middle | - | 20.39 | 88.62 | 78.7 |
| Panama | 4 302 037 | 8 | 19 202 | 12 531 | 326 | 0 | Upper middle | 56 000 | 5.05 | 59.76 | 82.41 |
| Algeria | 43 705 292 | 6 | - | 9 267 | 646 | 174 | Upper middle | - 50 002 | 0.00 | 81.09 | 92.13 |

^1^Estimates as at 2018; ^2^Estimates as at 2018; ^3^Estimates as at 11 June 2020; ^4^Estimates as at 31 May 2020; ^4^Estimates as at 31 May 2020; ^5^Estimates as at 31 May 2020; ^6^Estimates per billion as at 2018; ^7^Estimates as at 2018; ^8^Estimates as at 2017
