# Supplementary material for: An ecological study to evaluate the association of Bacillus Calmette-Guerin (BCG) vaccination on cases of SARS-CoV2 infection and mortality from COVID-19
Source: PLoS One. 2020 Dec 17;15(12):e0243707. doi: 10.1371/journal.pone.0243707 (PMC7746266; doi:10.1371/journal.pone.0243707)
Supplement: S2 Table — (DOCX) [file pone.0243707.s002.docx]

**S2 Table: Morbidity and mortality from SARS-CoV2 pandemic and the population, economic, and health characteristics of selected countries with a previous BCG policy**

| Country | **POPULATION INDICATORS** | | **COVID-19 INDICATORS** | | | **ECONOMIC INDICATORS** | | | **HEALTH INDICATORS** | | |
| --- | --- | --- | --- | --- | --- | --- | --- | --- | --- | --- | --- |
|  | Population size^1^ | Population >65 years^2^ (%) | Tests per capita^3^ (n) | Confirmed cases^4^ (n) | Fatal cases^5^ (n) | Gross domestic product ($)^6^ | Income level^7^ | Nett Immigration^8^ | Smoking prevalence rate | Stringency levels at 100 cases | Stringency levels 28 days after 10^th^ fatal case |
| Italy | 60 473 254 | 23 | 72 459 | 232 664 | 33 340 | 2 084 | High | 744 713 | 23.18 | 56.50 | 91.67 |
| Germany | 83 743 088 | 21 | 56 036 | 181 482 | 8 500 | 398 | High | 2 719 112 | 29.17 | 25.80 | 73.15 |
| France | 65 243 470 | 20 | 21 215 | 148 436 | 28 717 | 2 778 | High | 182 636 | 26.24 | 21.80 | 90.74 |
| United Kingdom | 67 819 564 | 18 | 89 037 | 272 830 | 38 376 | 2 855 | High | 1 303 250 | 16.59 | 11.11 | 75.93 |
| Switzerland | 8 642 376 | 19 | 51 049 | 30 762 | 1 656 | 705 | High | 259 999 | 21.40 | 27.38 | 76.85 |
| Austria | 8 997 504 | 19 | 56 238 | 16 638 | 668 | 455 | High | 324 998 | 33.01 | 16.93 | 69.44 |
| Sweden | 10 087 429 | 20 | 32 192 | 37 113 | 4 395 | 556 | High | 200 000 | 17.55 | 0.01 | 37.96 |
| Denmark | 5 788 234 | 20 | 129 417 | 11 633 | 571 | 356 | High | 75 998 | 13.15 | 46.69 | 74.07 |
| Norway | 5 412 827 | 17 | 50 362 | 8 411 | 236 | 434 | High | 140 000 | 18.00 | 11.11 | 66.67 |
| Czechoslovakia | 10 705 902 | 19 | 44 717 | 9 230 | 319 | 245 | High | 110 057 | 32.62 | 53.46 | 61.11 |
| Finland | 5 538 941 | 22 | 38 247 | 6 826 | 316 | 277 | High | 70 000 | 18.61 | 24.07 | 65.74 |
| Slovenia | 2 078 938 | 20 | 41 876 | 1 473 | 108 | 54 | High | 9 999 | 18.70 | 33.40 | 86.11 |
| Slovakia | 5 459 232 | 16 | 35 437 | 1 521 | 28 | 106 | High | 7 423 | 27.24 | 82.14 | 73.15 |

^1^Estimates as at 2018; ^2^Estimates as at 2018; ^3^Estimates as at 11 June 2020; ^4^Estimates as at 31 May 2020; ^4^Estimates as at 31 May 2020; ^5^Estimates as at 31 May 2020; ^6^Estimates per billion as at 2018; ^7^Estimates as at 2018; ^8^Estimates as at 2017
