# Supplementary material for: An ecological study to evaluate the association of Bacillus Calmette-Guerin (BCG) vaccination on cases of SARS-CoV2 infection and mortality from COVID-19
Source: PLoS One. 2020 Dec 17;15(12):e0243707. doi: 10.1371/journal.pone.0243707 (PMC7746266; doi:10.1371/journal.pone.0243707)
Supplement: S3 Table — (DOCX) [file pone.0243707.s003.docx]

**S3 Table: Morbidity and mortality from 2020 COVID-19 pandemic and the population, economic, and health characteristics of selected countries with no BCG policy**

| Country | **POPULATION INDICATORS** | | **COVID-19 INDICATORS** | | | **ECONOMIC INDICATORS** | | | **HEALTH INDICATORS** | | |
| --- | --- | --- | --- | --- | --- | --- | --- | --- | --- | --- | --- |
|  | Population size^1^ | Population >65 years^2^ (%) | Tests per capita^3^ (n) | Confirmed cases^4^ (n) | Fatal cases^5^ (n) | Gross domestic product ($)^6^ | Income level^7^ | Nett Immigration^8^ | Smoking prevalence rate | Stringency levels at 100 cases | Stringency levels 28 days after 10^th^ fatal case |
| Australia | 25 443 983 | 16 | 67 152 | 7 185 | 103 | 1 434 | High | 791 229 | 12.65 | 19.44 | 69.44 |
| New Zealand | 4 814 777 | 16 | 60 351 | 1 154 | 22 | 205 | High | 74 403 |  | 79.63 | 36.11 |
| Spain | 46 755 531 | 19 | 95 507 | 239 600 | 29 043 | 1 419 | High | 200 000 | 26.83 | 11.11 | 85.19 |
| Belgium | 11 580 318 | 19 | 84 895 | 58 186 | 9 453 | 543 | High | 240 000 | 21.50 | 13.89 | 81.48 |
| Netherlands | 17 127 688 | 19 | 26 041 | 46 257 | 5 951 | 914 | High | 80 000 | 22.50 | 3.97 | 79.63 |
| Israel | 8 630 359 | 12 | 76 858 | 17 012 | 284 | 371 | High | 50 002 | 29.85 | 40.49 | 87.96 |
| Cyprus | 1 205 786 | 14 | 111 616 | 943 | 17 | 25 | High | 25 000 |  | 94.71 | 94.44 |
| Bahrain | 1 691 726 | 2 | 232 066 | 10 793 | 17 | 38 | High | 239 000 | 46.44 | 25 |  |
| United States of America | 330 634 118 | 16 | 68 386 | 1 716 078 | 101 567 | 20 544 | High | 4 774 029 | 15.16 | 8.33 | 72.69 |
| Canada | 37 679 750 | 17 | 52 732 | 89 741 | 6 996 | 1 713 | High | 1 210 159 | 12.10 | 21.83 | 72.69 |
| Lebanon | 6 825 240 | 7 | 14 580 | 1 191 | 26 | 57 | Upper middle | -150 060 | 42.15 | 62.57 | 81.48 |

^1^Estimates as at 2018; ^2^Estimates as at 2018; ^3^Estimates as at 11 June 2020; ^4^Estimates as at 31 May 2020; ^4^Estimates as at 31 May 2020; ^5^Estimates as at 31 May 2020; ^6^Estimates per billion as at 2018; ^7^Estimates as at 2018; ^8^Estimates as at 2017
